# Intermedin protects HUVECs from ischemia reperfusion injury via Wnt/β-catenin signaling pathway

**DOI:** 10.1080/0886022X.2019.1587468

**Published:** 2019-04-01

**Authors:** Yanhong Wang, Zhijing Wu, Jihua Tian, Yang Mi, Xiaojun Ren, Jing Kang, Wan Zhang, Xiaoshuang Zhou, Guiqin Wang, Rongshan Li

**Affiliations:** a Department of Microbiology and Immunology, Shanxi Medical University, Taiyuan, China;; b Department of Nephrology, The Affiliated People’s Hospital of Shanxi Medical University, Shanxi Provincial People’s Hospital, Shanxi Kidney Disease Institute, Taiyuan, China;; c Department of Urology, First Hospital of Shanxi Medical University, Taiyuan, China;; d Department of Nephrology, Shanxi Dayi Hospital of Shanxi Medical University, Taiyuan, China

**Keywords:** IMD, angiogenesis, Wnt/β-catenin signaling, hypoxia/reoxygenation

## Abstract

Intermedin (IMD) is a member of the calcitonin gene-related peptide (CGRP) superfamily and a pro-angiogenic factor. In the present study, we identified activation of the Wnt/β-catenin signaling pathway by IMD. Adding CoCl_2_ HUVECs was used to establish an *in vitro* model. The migration of HUVECs was measured by wound healing assays and transwell migration assays. Capillary formation was measured using tube formation assays. Immunocytochemistry (ICC) analysis was used to evaluate VEGF and RAMP2 expression in HUVECs. The relevant signaling molecules were detected with western blot. Our study shows that IMD could promote H/R impaired HUVECs migration and tube formation *in vitro*. On the other hand, inhibition of Wnt/β-catenin signaling led to the suppression of this promotion of migration and tube formation. This result suggests that Wnt/β-catenin signaling is correlated to IMD induced angiogenesis. Analysis of results from ICC assays indicated that IMD works through increasing levels of VEGF and RAMP2. Meanwhile, the Wnt/β-catenin signaling specific inhibitor IWR-1-endo was shown to down-regulate VEGF and RAMP2 expression. Western blot results further confirmed the signaling mechanism by which IMD promotes angiogenesis. Thus, Wnt/β-catenin signaling plays an important role in IMD induced neovascularization. The data further suggest that the PI3K axis contributes positively downstream.

## Introduction

Renal ischemia-reperfusion injury (IRI) plays an important role in ischemic acute renal failure (ARF), which carries high morbidity and mortality following kidney transplantation and shock. Few effective therapeutic options exist to treat ARF [1–3]. The kidney is a hypertransfusion organ, making it more susceptible to IRI. This type of injury happens to acutely ischemic tissue when blood flows back into it. IRI may cause further damage to surrounding tissue, including injury of renal tubular-epithelial cells and damage to vasculature around tubules [4]. Therefore, protecting vessels against IRI, promoting endothelial cell repair, stimulating angiogenesis is absolutely necessary for the treatment of ARF. The canonical Wnt pathway is critical for signaling in the development of vascular system [5]. As a co-transcription factor, β-catenin can activate canonical Wnt signaling when it accumulates in the nucleus binding target genes. Otherwise, β-catenin will be degraded by the Axin/APC/GSK3β/CKI complex [6]. It has been reported that the Wnt/β-catenin pathway can induce vascular endothelial growth factor (VEGF) to promote neovascularization [7,8].

Intermedin (IMD), also known as adrenomedullin-2 (AM2), is a recently identified peptide that belongs to the calcitonin gene-related peptide (CGRP) family [9,10]. Recent studies have suggested that IMD distributed in the cardiovascular and renal systems could act as an endogenous protective peptide under hypoxia and reoxygenation (H/R) conditions [11–14]. Our team previously discovered that IMD can protect tubular cells from IRI by way of promoting proliferation, increasing cyclin D1 expression, and inhibiting endoplasmic reticulum stress *in vitro* [15,16]. Several studies have shown that IMD participates in the regulation of angiogenesis via ERK, PI3K-Akt-eNOS-NO, cAMP/PKA and VEGF signaling pathways [17–19].

However, no evidence elucidates the connection between IMD and canonical Wnt signaling during neovascularization. In this study, we identify the mechanism of IMD under H/R conditions.

## Materials and methods

### Cells and reagents

Human umbilical vein endothelial cells (HUVECs) was obtained from the Cell Bank of the Chinese Academy of Sciences (Shanghai, China) and cultured in DMEM (HyClone, SH30022.01) with 10% fetal bovine serum (FBS; Solarbio, F8245). Cell cultures were kept in an incubator maintained at 37 °C in a humidified atmosphere with 5% CO_2_. Intermedin/adrenomedullin-2 (IMD; Rat, Phoenix Pharmaceuticals Inc) treatment was prepared by adding IMD into DMEM for a final concentration of 20 ng/ml [17]. Inhibition of the Wnt/β-catenin signaling pathway was performed by adding IWR-1-endo (MCE, 1127442-82-3) into DMEM to a final concentration of 10 μM.

### HUVECs hypoxia/reoxygenation (H/R) model and experiment design

A chemical model of hypoxia was established with HUVECs by adding cobalt chloride (CoCl_2_) in DMEM. Reoxygenation was achieved by washing with phosphate-buffered saline (PBS) 3 times and changing the medium to DMEM for 1 h in an incubator. The experiment was divided into groups as follow: untreated HUVECs in serum-free culture medium (normal); H/R model (H/R); HUVECs preincubated with IMD for 1 h at 37 °C then changed to culture medium as for the H/R group (H/R + IMD); HUVECs preincubated with IWR-1-endo in incubator for 1 h, then changed to culture medium as for the IMD + H/R group with IWR-1-endo added (H/R + IMD + IWR).

### MTT assay

The viability of cells in the H/R model was assessed using the 3-(4,5-dimethylthiazol-2-yl)-2,5-diphenyl tetrazolium bromide (MTT) assay. HUVECs were incubated in 96-well culture plates (Corning Inc, Corning, NY) overnight at a concentration of 1 × 10^4^ cells/well in sextuplicate. In preliminary experiments, we identified a range in which the model showed a better dose-dependent relationship after 24 h of hypoxia and 1 h reoxygenation. As a next step, we worked to ensure the proper concentration to build a stable H/R model. So, a series of concentrations of CoCl_2_ (0–2.4 mM) were added into the serum-free culture medium. After 24 h of hypoxia and 1 h reoxygenation, 20 μl of 5 mg/ml MTT (Solarbio, M8180, China) was added into each well, followed by incubation for 4 h in a condition protected from light. Then, the cell supernatant was discarded, and 150 µl dimethyl sulfoxide (DMSO) was administered with gently shaking for 10–15 min. The absorbance was recorded at 490 nm with a Microplate Spectrophotometer (C-5000, Institute of Biophysics, Chinese Academy of sciences, China).

### Wound healing assays

HUVECs were seeded in 6-well culture plates (Corning Inc, Corning, NY), then incubated. After reaching 70–80% confluence, cell monolayers were scratched with a sterile plastic tip (10 μl). Then, they were cultured in FBS-free culture medium, washed with PBS 3 times, then subjected to various treatments as indicated in the description of the four experimental groups. At 0 h and 24 h following scratching of the monolayers, cell migration in five randomly selected fields was observed using an optical microscope (×100 magnification). Images were captured using a digital camera (Olympus C-5060, Japan).

### Transwell migration assays

Cell migration was assessed using a transwell migration assay. HUVECs were harvested from each group. 2 × 10^4^ cells/well in 200 µl DMEM medium supplemented with 1% FBS were placed in the upper chamber (pore size, 8 µm, Corning Inc). 24-Well culture plates (Corning Inc, Corning, NY). The lower chamber was filled with medium containing 10% FBS (600 µl). After 24 h migration, the cells that had not migrated on the upper surface of the membrane were scraped gently with a cotton swab. The migrated cells were fixed in 95% ethyl alcohol for 15–20 min and stained with 0.1% crystal violet for 15 min, then washed three times with PBS. Cells were counted in three randomly selected fields under an optical microscope (×200 magnification) and photographed using a digital camera (Olympus C-5060, Japan).

### HUVECs tube formation assay

96-Well plates were pre-chilled. Each well was coated with 50 µl of 50% Matrigel (Solarbio, M8370, China), which was polymerized for 30 min at 37 °C. HUVECs were harvested from each group (3 × 10^4^ cells/well) in 100 µl DMEM medium supplemented with 1% FBS. These aliquots of cell cultures were added to each well and incubated at 37 °C, 5% CO_2_ for 12 h. Tube formation was observed using a light microscope (×100 magnification) and photographed with a digital camera (Olympus C-5060, Japan).

### Immunocytochemistry (ICC) analysis

Cells attached to coverslips were fixed in 4% paraformaldehyde for 10–15 min. Then, they were permeabilized for 10 min with 0.5% Triton X-100. Next, we used Histostain^TM^-Plus Kits (Bioss, SP-0023, China), following the manufacturer’s instructions, for visualization using diaminobenzidine (DAB). Cells were then counterstained with Mayer' Hematoxylin (Solarbio, G1080, China) to label the nuclei. The primary antibodies used are as follows: VEGF (1:100; Bioss, bs-0279R, China); RAMP-2 (1:100; Bioss, bs-11971R, China). Slides were observed using a light microscope (×100 magnification) and photographed with a digital camera (Olympus C-5060, Japan).

 

### Reverse transcription polymerase chain reaction and real-time PCR

The method of RT-PCR was described in our previous work [16]. Real-time PCR amplification was carried out using the SYBR Green I systemtheStrategene M3000 Sequence Detection System (Stratagene). RAMP2 was amplified by primers: 5′-GGGACGGTGAAGAACTATGA-3′ and 5′-AAGCCCAGGTCAAACAACTC-3′; VEGF was amplified by primers: 5′-GCGGATCAAACCTCACCAAG-3′ and 5′-GCTTTCGTTTTTGCCCCTTTC-3′; β-actin was amplified by primers: 5′-TGGACTTCGAGCAAGAGATG-3′and 5′-TGTTGGCGTACAGGTCTTTG-3′.

### Western blot analysis

HUVECs from each group were added in RIPA lysis buffer (Beyotime, P0013B, China). Protein concentrations were measured using a BCA Protein Assay kit (KeyGEN Biotech, China). Proteins were separated on 10% SDS-polyacrylamide gels assayed by immunoblot using β-catenin antibody (Cell Signaling Technology, 8480S, USA), p-β-catenin (Ser675) antibody (Cell Signaling Technology, 9567S, USA), GSK3β (Ser9) antibody duet (Cell Signaling Technology, 8213S, USA), and β-actin antibodies (ZSGB-Bio, PR-0255, China). Secondary antibody used was (HRP)-conjugated goat anti-rabbit or anti-mouse IgG (ZSGB-Bio, ZB-2305, ZB-2301, China) at 1:5000. Signals were visualized with super enhanced chemiluminescence (ECL) detection reagent (BOSTER, AR1172, China) and detected by Quantity One analysis system (Bio-Rad).

### Statistical analysis

Photographs were analyzed using Image J software and Image pro-plus 6.0 (Media Cybernetics, Rockville, MD, USA). Data are presented as mean ± SD. The statistically significant differences were identified by one-way analysis of variance (ANOVA), followed by LSD test. Values of *p* < .05 were considered statistically significant.

## Results

### Cellular viability of CoCl_2_ treated HUVECs

**Figure 1.**
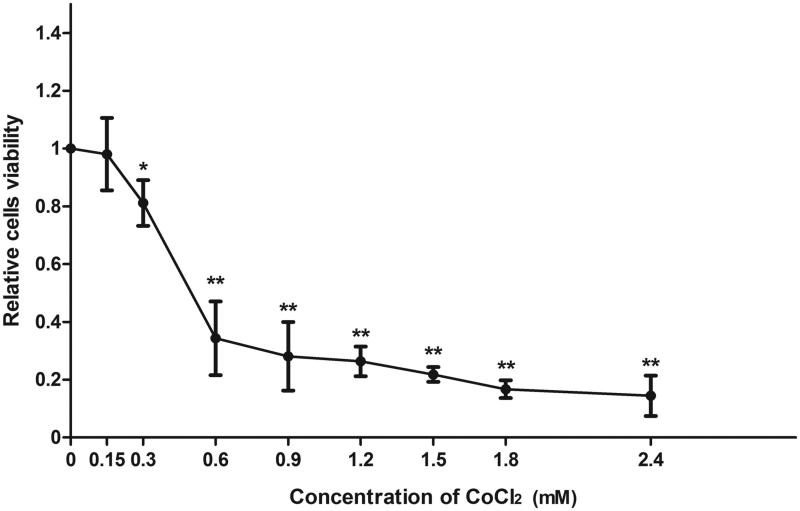
CoCl_2_ treatment induced HUVECs chemical hypoxia. Viability was assessed via MTT assay using different concentrations of CoCl_2_ (0–2.4 μM) in 9 groups. Data are displayed as percentage of positive cells compared with control. Each bar represents the mean ± standard deviation (SD), (*n* = 3, *p < .05, **p < .01 vs. control).

HUVECs were incubated with CoCl_2_ to establish a stable H/R model *in vitro* to mimic the pathological process of IRI. The MTT assay was used to determine the proper dose of CoCl_2_. As shown in Figure 1, the viability of HUVECs decreased significantly with 300 μM CoCl_2_ (*p* < .05). This condition was used for the following experiments.

### IMD Promotes HUVEC migration and tube formation through the Wnt/β-catenin signaling pathway

**Figure 2.**
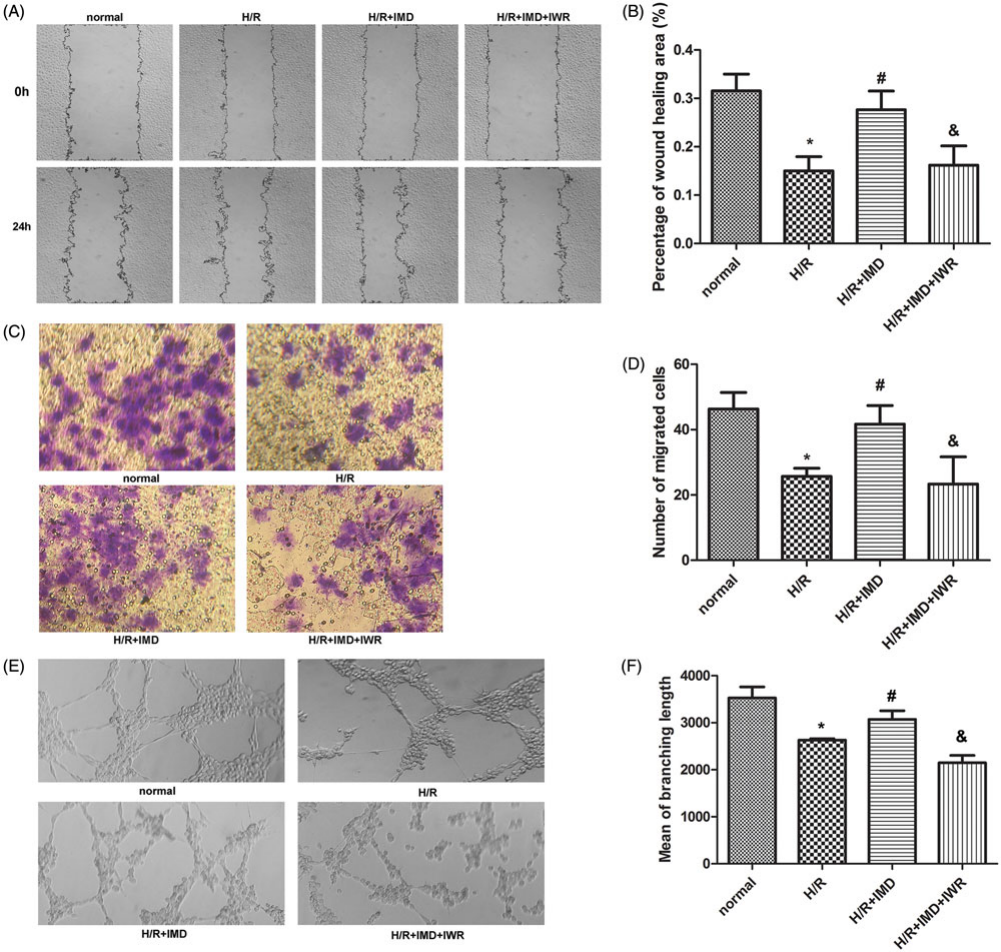
IMD promotes HUVEC migration and tube formation *in vitro*. (A) HUVECs in each group were scraped with 10 μl sterile plastic tips, then the images were captured at 0 and 24 h (×100 magnification). (B) Histogram shows the percentage of wound healing area (*n* = 5). Wound areas were determined by Image J. (C) Transwell migration assays were photographed after 24 h using an optical microscope (×200 magnification). (D) Histogram shows the number of migrated cells (*n* = 3). [In (B,D) *p < .01 vs. control, #*p* < .01 vs. H/R group, &*p* < .01 vs. H/R + IMD group]. (E) After 12 h HUVECs formed network structures on matrigel coated plates. Structures were observed using a light microscope (×100 magnification). (F) Histogram shows the mean of branching length in each group (*n* = 3, *p < .01 vs. control, #*p* < .05 vs. H/R group, &*p* < .01 vs. H/R + IMD group). Each bar represents the mean ± SD.

The wound healing assay and transwell migration assay were used to assess the migration capability of HUVECs. By comparing the wound area among groups (Figure 2(A,B)), we observed that H/R inhibits HUVEC movement. On the other hand, we observed that IMD effectively promotes cell migration. IWR-1-endo was observed to suppress IMD induced protection at a concentration of 10 μM. Moreover, in a transwell migration assay, the number of HUVECs that migrated to the other side was lower in the H/R model. On the other hand, IMD strongly enhanced cell movement, increasing migrated cell count. When the specific inhibitor IWR-1-endo blocked the canonical Wnt pathway, the aforementioned effect was suppressed (Figure 2(C,D)). To further investigate whether IMD could alter tube formation of HUVECs exposed to H/R, we first collected cells from each group and incubated them in the matrigel coated plates. After 12 h’ incubation, HUVECs may form network structures. Results showed in Figure 2(E,F) indicate that IMD increased tube formation in the H/R model, and pretreatment with IWR-1-endo reduced the network formation.

### Comparison of cytokines expression in each group

**Figure 3.**
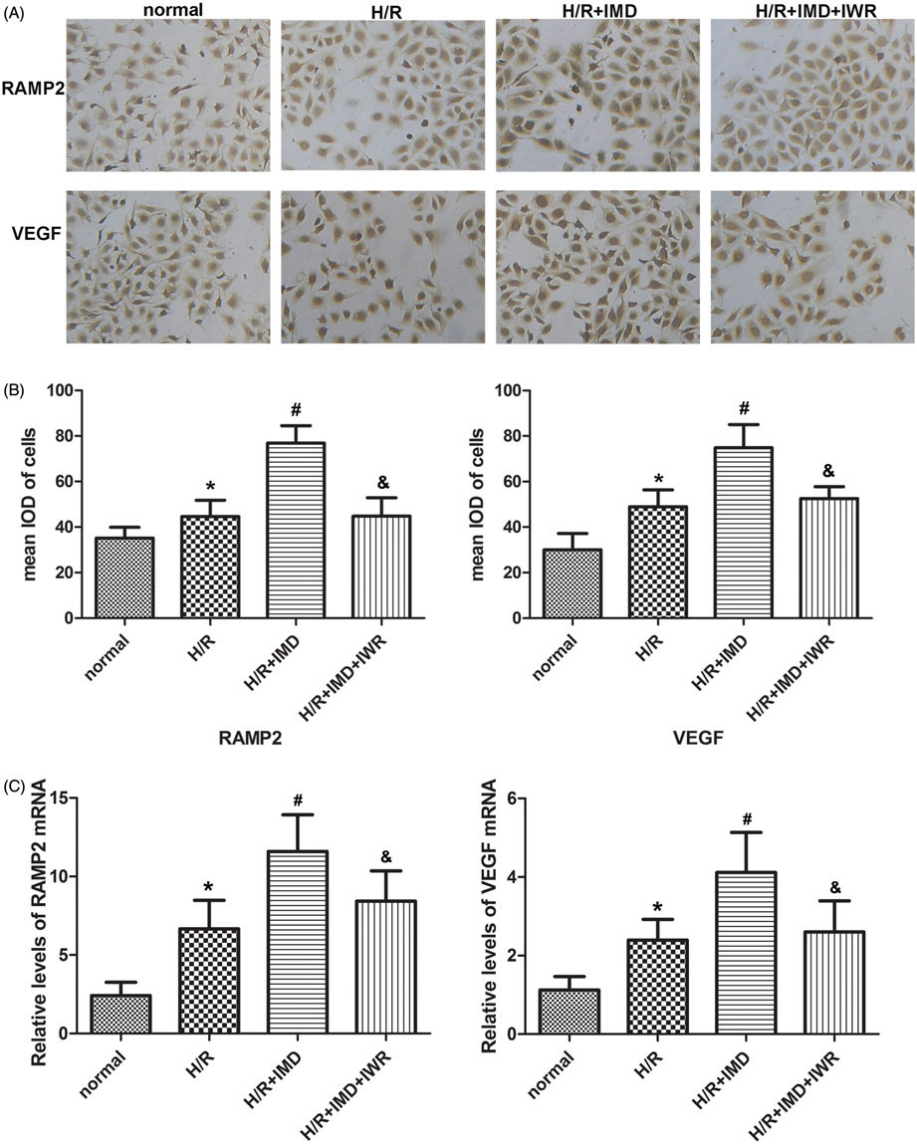
IMD influenced the expression of vascular molecules. (A) HUVECs were stained with RAMP2 antibody and VEGF antibody, then visualized by DAB under a light microscope (×100 magnification). (B) Histogram shows the mean IOD of cells. Each bar represents the mean ± SD (*n* = 5, *p < .01 vs. control, #*p* < .01 vs. H/R group, &*p* < .01 vs. H/R + IMD group). (C) Histogram shows the relative levels of RAMP2 and VEGF mRNA in each group. Each bar represents the mean ± SD (*n* = 5, *p < .01 vs. control, #*p* < .01 vs. H/R group, &*p* < .01 vs. H/R + IMD group).

In order to elucidate the mechanism of the pro-angiogenic effect induced by IMD, we performed an ICC assay. This assay was used to test the expression of VEGF and IMD acceptor RAMP2. HUVECs treated with IMD expressed more VEGF and RAMP2 than did the H/R group. However, H/R cells treated with IMD and IWR-1-endo showed no increase in levels of VEGF and RAMP2. The mRNA expression levels of RAMP2 and VEGF in each group were measured by RT-PCR. Compare with the HR group, treatment with IMD significantly increased the mRNA expression of RAMP2 and VEGF. In the inhibitor group, IWR-1-endo initiate the degradation of RAMP2 and VEGF mRNA (Figure 3).

### The expression of Wnt/β-catenin signaling pathway-related proteins

**Figure 4.**
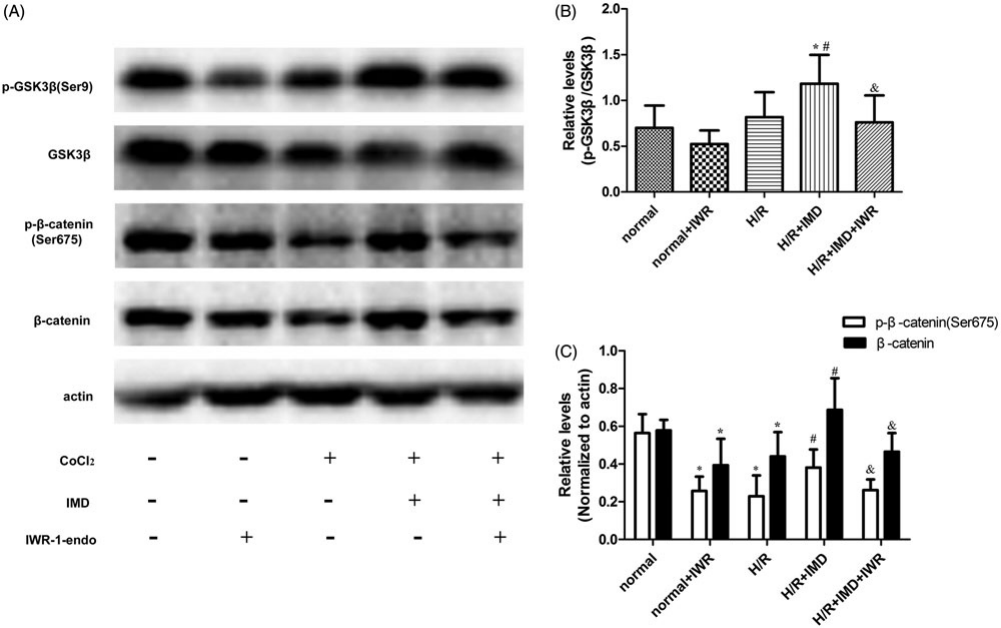
IMD upregulated the Wnt/β-catenin signaling pathway. (A) Western blot of β-catenin, p-β-catenin (Ser675), GSK3β, GSK3β (Ser9), β-actin expression from each group. (B,C) Histogram shows the relative level of proteins, IWR-1-endo can specifically inhibit the level of β-catenin without changing the GSK3β expression. Results are expressed as the mean ± SD (*n* = 8, *p < .01 vs. control, #*p* < .01 vs. H/R group, &*p* < .05 vs. H/R + IMD group).

To explore the mechanism of benefits induced by IMD, we evaluated crucial proteins of the Wnt/β-catenin signaling pathway in each group (Figure 4(A)). Western blot revealed that the expression of β-catenin and p-β-catenin (Ser675) were substantially lower in the H/R model than in the negative control. In contrast, when H/R injured cells were treated with IMD, levels of β-catenin and p-β-catenin (Ser675) were significantly higher than those in H/R cells and H/R cells treated with a combination of IMD and IWR-1-endo to IMD (Figure 4(C)). We also observed the changes of GSK3β and p-GSK3β (Ser9). The level of p-GSK3β (Ser9) was higher in H/R cells treated with IMD than in H/R cells without treatment and higher than in H/R cell incubated with a combination of IMD and IWR-1-endo (Figure 4(B)).

## Discussion

Kidney is especially sensitive to IRI. The pathological process of IRI includes tubular cell injury and peritubular capillary damage. IMD has broad biological effects. Our study explores the mechanism by which IMD, serving as a pro-angiogenic factor, protects renal tubular epithelial cells from IRI [17]. Through this mechanism, promoting angiogenesis may be a novel strategy for ischemic ARF therapy. The Wnt/β-catenin signal pathway is highly conserved. This pathway is widely known for its role in angiogenesis and vessel morphogenesis [5]. However, no previous reports show that IMD can induce Wnt/β-catenin signaling to promote these processes.

In the present study, we used HUVECs to model neovascularization *in vitro*. We established four different condition groups: normal, H/R, H/R + IMD, H/R + IMD + IWR-1-endo. Then, we observed differences in these groups. We found that in the H/R group, the migration ability of HUVECs were lower than in the normal group. Migration ability was restored with the addition of IMD. This result was in agreement with that of the *in vitro* tube formation assay, a representative test of neovascularization. Interestingly, the Wnt/β-catenin signaling inhibitor IWR-1-endo attenuated the protection of IMD. In neovascularization, many cytokines are produced that work together to manage this physiological process. VEGF is a potent pro-angiogeneic factor with strong abilities to promote proliferation, permeability and chemotaxis. The expression of VEGF is sensitive to β-catenin/TCF [7,20,21]. High levels of VEGF causes vascular irregularities and increase vessel permeability. However, IMD can restrict excessive vessel formation and reduce HUVECs permeability [18,22]. We measured the expression levels of RAMP2, a receptor of IMD expressed on vascular endothelial cells participating in angiogenesis [10,23]. We confirmed that β-catenin is involved in the regulation of RAMP2, which may synergistically increase HUVEC reaction to IMD.

Further, we investigated the molecular mechanism of Wnt/β-catenin signaling activated by IMD. GSK-3β is a component of the β-catenin destruction complex which mediates β-catenin degradation. Wnt signaling activity inactivation can lead to β-catenin accumulated in the cytoplasm [6]. Cytosolic β-catenin is a key point for Wnt/β-catenin signaling. β-catenin can be transferred into nucleus binding a target gene when phosphorylated at serine 675 [24]. In our study, we observed that compared with the H/R impaired group, the IMD treated H/R group had higher levels of β-catenin and p-β-catenin (Ser675). Blocking Wnt/β-catenin signaling reduced these effects induced by IMD. This result indicates that IMD promotes Wnt signaling through (1) enhancing β-catenin transcription, (2) improving the phosphorylation level of β-catenin at site Ser675. Recent studies suggested that IMD stimulates cAMP/PKA signaling [10]. β-catenin and GSK-3β have phosphorylation sites for PKA at Ser675 and Ser9, respectively [24–26]. Therefore, there might exist crosstalk between Wnt/β-catenin and cAMP/PKA signaling. As shown in Figure 4(C), IMD treatment corresponded with an increase in the level of p-GSK-3β (Ser9). However, IWR-1-endo specifically inhibits the canonical Wnt pathway without changing the expression of p-GSK-3β (Ser9). Surprisingly, we discovered an inconsistent result in the H/R + IMD + IWR-1-endo group. Earlier studies suggest that there are at least two different pools of GSK-3β: one with AXIN mediated degradation of β-catenin and resistant to phosphorylation at Ser9 or Ser21; another under the control of PI3K/Akt signaling [7,27,28]. Thus, we inferred that Wnt/β-catenin signaling is upstream of PI3K/Akt signaling, consistent with reports from a former study [7].

Although our current study provided significant results, there are still some limitations. Firstly, the mechanism of neovascularization is very complicated; some details of crosstalk interaction are still obscured in ambiguity whose resolution requires further research. Secondly, this study only analyzed the protection of exogenic IMD, while the decrease of endogenic IMD could be another interesting point. Thirdly, the further experiment *in vivo* we intended to do as an independent part may help us to get much more evidence. In conclusion, our study revealed that exogenous IMD up-regulates the expression of VEGF and RAMP2 at least partially via activating Wnt/β-catenin signaling, finally promoting angiogenesis of H/R impaired HUVECs *in vitro*.
